# Basic helix-loop-helix (*bHLH*) gene family in rye (*Secale cereale* L.): genome-wide identification, phylogeny, evolutionary expansion and expression analyses

**DOI:** 10.1186/s12864-023-09911-3

**Published:** 2024-01-17

**Authors:** Xingyu Chen, Caimei Yao, Jiahao Liu, Jintao Liu, Jingmei Fang, Hong Deng, Qian Yao, Tairan Kang, Xiaoqiang Guo

**Affiliations:** 1 Sichuan Industrial Institute of Antibiotics, School of Pharmacy, Chengdu University, Chengdu, 610106 PR China; 2 School of Food and Biological Engineering, Chengdu University, Chengdu, 610106 PR China

**Keywords:** Rye, *bHLH* gene family, Genome-wide analysis

## Abstract

**Background:**

Rye (*Secale cereale*), one of the drought and cold-tolerant crops, is an important component of the Triticae Dumortier family of Gramineae plants. Basic helix-loop-helix (*bHLH*), an important family of transcription factors, has played pivotal roles in regulating numerous intriguing biological processes in plant development and abiotic stress responses. However, no systemic analysis of the bHLH transcription factor family has yet been reported in rye.

**Results:**

In this study, 220 *bHLH* genes in *S. cereale* (*ScbHLHs*) were identified and named based on the chromosomal location. The evolutionary relationships, classifications, gene structures, motif compositions, chromosome localization, and gene replication events in these *ScbHLH* genes are systematically analyzed. These 220 *ScbHLH* members are divided into 21 subfamilies and one unclassified gene. Throughout evolution, the subfamilies 5, 9, and 18 may have experienced stronger expansion. The segmental duplications may have contributed significantly to the expansion of the *bHLH* family. To systematically analyze the evolutionary relationships of the *bHLH* family in different plants, we constructed six comparative genomic maps of homologous genes between rye and different representative monocotyledonous and dicotyledonous plants. Finally, the gene expression response characteristics of 22 *ScbHLH* genes in various biological processes and stress responses were analyzed. Some candidate genes, such as *ScbHLH11*, *ScbHLH48*, and *ScbHLH172*, related to tissue developments and environmental stresses were screened.

**Conclusions:**

The results indicate that these *ScbHLH* genes exhibit characteristic expression in different tissues, grain development stages, and stress treatments. These findings provided a basis for a comprehensive understanding of the *bHLH* family in rye.

**Supplementary Information:**

The online version contains supplementary material available at 10.1186/s12864-023-09911-3.

## Background

The basic helix-loop-helix (*bHLH*) transcription factor family is the second largest transcription factor family after *MYB* in plants, which is widely present in eukaryotes [1]. The *bHLH* transcription factor was first discovered in animals [2]. The molecular architecture of the *Lc* gene within the maize R locus, responsible for encoding proteins comprising 610 amino acids, was elucidated through cDNA sequencing. This intricate mechanism governs the biosynthesis of anthocyanins, representing the first reported instance of a plant-based *bHLH* gene [3]. The domain of the bHLH protein consists of two parts with different functions, namely the basic region and the helix-loop-helix region (HLH) [4, 5]. Among them, the basic domain is located in the N-terminal of bHLH proteins, with about 15 ~ 20 amino acids. This region is diversified in general, except for some amino acid residue sites. These conserved amino acid residues, namely E9 and R13, play a crucial role in conferring the binding capacity of bHLH proteins to specific cis-elements such as E-box (CANNTG) and G-box (CACGTG) elements [6, 7]. Moreover, the HLH region, comprising approximately 40 amino acids, is situated at the C-terminal of the bHLH proteins. The HLH region consists of two alpha helixes and a connecting region (loop) of variable length. Five amino acid residues in the HLH region (Leu23/64, Leu/Iso54, Val61) and two amino acid residues in the ring region (Lys/Arg47) are highly conserved in the plant *bHLH* genes [8–12]. Most bHLH proteins perform their biological functions by forming dimers. For example, Brownlie et al. [13] reported that homodimers and heterodimers of bHLH proteins can compete for a common DNA target site (E-box), where Leu23/54 in the HLH region is an essential amino acid residue for dimer formation. Ciarapica et al. [14] suggested that the dimer function of plant bHLH proteins were determined by the formation of molecular scaffolds, affinity interfaces between key amino acids involved in dimer molecular recognition in conserved HLH domains, and their interactions with chaperone proteins.

In animals, bHLH proteins are classified into six major groups (from A to F), according to their DNA-binding specificities, sequence homology, and structural features, which are in turn divided into smaller subfamilies. Each group contains subfamilies of bHLH proteins that have highly conserved DNA-binding and protein-protein interaction domains [15, 16]. At present, the classification of bHLH transcription factors in different plants is still uncertain. Pires and Dolan [17] used transcription factor sequences of the bHLH proteins of nine terrestrial plant and algal species to classify their evolutionary relationships, dividing these members into 26 subfamilies. Among them, 23 subfamilies contained the bHLH proteins of both plants, which were lycopods and angiosperms. Twenty subfamilies were identified as descendants of the common ancestor that gave rise to both mosses and vascular plants. On the other hand, six subfamilies within the vascular plants’ group do not include any mosses. These six subfamilies have continued to evolve and diversify since their separation from the ancestral group that included both mosses and vascular plants. In model plants, phylogenetic analysis of 147 *bHLH* genes divided these members into 21 subfamilies in *Arabidopsis thaliana* [11]. The 167 *OsbHLH* TFs have been divided into 22 subfamilies in rice [7]. The *bHLH* genes have been identified in many plants, including *Brassica rapa* subsp. *pekinensis* [18], *Solanum lycopersicum* [19], *Phaseolus vulgaris* [20], *Malus domestica* [21], *Arachis hypogaea* [22], *Brachypodium distachyon* [23], *Zea mays* [24], *Triticum aestivum* [25], *Phyllostachys edulis* [26], *Ziziphus jujuba* [27], *Capsicum annuum* [28], *Panax ginseng* [29], *Ananas comosus* [30], *Fagopyrum tataricum* [31], *Sorghum bicolor* [32], *Setaria italica* [33], *Dactylis glomerata* [34], *Brassica oleracea* var. *botrytis* [35], and *Jatropha curcas* [36]. These *bHLH* genes are mainly involved in the defense response of plants to abiotic stresses such as high temperature, cold, drought, and elevated salinity. Most of these stresses are unique to terrestrial plants. Therefore, genome-wide analysis of the *bHLH* family will provide a useful aid to comprehend the adaptation and evolution of different plants in their environments.

The *bHLH* gene family is known to exist in organisms for a very long time. As early as 415 million years ago, *bHLH* transcription factors were present in the ancestors of angiosperms [37, 38]. Pires et al. [17] reported that these transcription factors evolved to include a series of highly conserved short amino acid prescriptions that were present in plants that existed 400 million years ago. However, the evolution of ancestral *bHLH* members into different subfamilies and the gene expansion induced by structural variation in different subfamilies is still unclear, which is speculated to be related to repetitive events, including segment and tandem duplications [31, 32]. The *bHLH* family of plants with monocotyledon amplifies members in a monophyletic manner, and this relationship may be established after its separation from the *bHLH* members of dicotyledon, several types of ancestors did not converge or evolve in parallel. Nevertheless, the origin of the affinity observed in the *bHLH* members of Triticeae Dumortier may be attributed to evolutionary clustering within the Gramineae family [39]. The interplay and evolutionary relationship between these members and other biological bHLH proteins remain shrouded in ambiguity. Therefore, examining the homologous *bHLH* genes in many Triticeae Dumortier plants is significant to analyze the relatives of members of different subfamilies and sort out the evolution and derivation of these bHLH proteins.

*bHLH* transcription factors play important roles in various metabolic and developmental processes in plants, such as light response, hormone signaling, fruit and seed development, and plant morphological development. For example, *SPATULA* was the first *bHLH* gene found in *A. thaliana* to regulate the development of carpel margin and inner pollen tissue, and also redundantly regulate the growth of stem tip meristem, leaves, stamens, and roots [40, 41]. The *LAX* gene, a *bHLH* member, can regulate the formation of the axillary bud primordium in rice [42]. *AtRGE1* (RETARDED GROWTH OF EMBRY01) is specifically expressed in the endosperm during embryonic development and controls seed growth in *A. thaliana* [43]. *SPEECHLESS* and two homologous genes are required for the initiation, proliferation, and terminal differentiation of cells in the stomatal lineage, ultimately leading to the formation of functional stomata on the leaf surface [44]. TabHLH123 interacts with TaMOR, an essential regulator of crown root initiation, to regulate stem base and root development of wheat, and participate in plant height building and auxin metabolism [45]. *OsbHLH98* exerts negative regulation on the leaf angle by modulating the quantity and dimensions of paraxial parenchyma cells at the leaf junction. Its involvement extends to plant morphogenesis and photosynthesis, presenting a multifaceted role in plant biology [46]. *bHLH* is also involved in response to various hormones. JAZ protein, bHLH (TTG8, GL3), and R2R3-MYB transcription factors (MYB75 and Glabra1) jointly formed WD-repeat/bHLH/MYB transcription complex, which regulated anthocyanin accumulation by inhibiting jasmonic acid content [47]. *CESTA* and its homologs induce the expression of the gibberellin 2-oxidase gene *GA2ox7* in *A. thaliana* are enhanced by brassinosteroids (BRs) [48]. *SlPRE2* regulates fruit development and plant response to gibberellin in tomatoes. When *SlPRE2* is silenced through genetic modification, the resulting plants exhibit reduced grain size, as well as thinner peels, smaller placentas, and smaller seeds [49]. In addition, the C-terminal domain (SD) of BIGPETALp promotes petal growth by interacting with ARF8 to participate in the auxin transport pathway and influence cell expansion in *Arabidopsis* [50].

More and more studies have revealed the importance of *bHLH* transcription factors in regulating the plant response to abiotic stresses such as drought, salinity, and high temperature. For example, *SlbHLH96* has been shown to act as a positive regulator of drought tolerance in tomatoes. Overexpression of *SlbHLH96* leads to enhanced drought tolerance by activating the expression of genes encoding antioxidants, ABA signaling molecules, and stress-related proteins, which ultimately leads to better stress-adaptive responses in the plant [51]. Overexpression *FtbHLH3* of Tartary buckwheat can improve the tolerance to drought stress by activating the antioxidant system and up-regulating the expression of various metabolic pathway genes in *A. thaliana* [52]. Overexpression of *PebHLH35* of *Populus euphratica* resulted in longer initial roots, more leaves, and increased tolerance to drought stress in *A. thaliana* by regulating stomatal density, stomatal opening, photosynthesis, and growth [53]. In sorghum, *SbbHLH85* significantly increases the number and length of root hairs through participation in ABA and auxin signaling pathways and leads to sodium ion absorption and improved resistance to salt stress [54]. *CabHLH035* regulates Na^+^ homeostasis and proline biosynthesis to enhance salt tolerance. It accomplishes this by binding to the *CaSOS1* and *CaP5CS* gene promoters and actively stimulating their expression in capsicum [55]. In wheat, *TabHLH1* reduces leaf water loss rate and increases proline and soluble sugar content, through osmotic regulation of substance accumulation and intracellular ROS homeostasis mediated by the ABA signaling pathway, thus contributing to drought and salt stress tolerance [56]. In a high-temperature environment, *SPEECHLESS* and *PIF4* (PHYTOCHROME-INTERACTING FACTOR 4) jointly mediated porosity development processes, modulating adaptation to stress through negative feedback [57]. Although progress has been made in the function and identification of *bHLH* transcription factors in several plant species, the specific physiological roles and regulatory mechanisms of many bHLH proteins in rye have been poorly reported.

Rye (*S. cereale*), an ancient and important cereal crop, is a monocotyledonous plant mainly cultivated in Europe, Asia, and Latin America [58]. Rye is the only crop rich in gluten protein next to wheat, which is widely used by the food industry around the world for making bread, biscuits, flour, drinks, beer, and other products [59]. Compared with wheat, rye possesses a very balanced distribution of amino acids, so it is considered to be an important dietary source [60, 61]. In addition, *S. cereale* is a diploid plant belonging to the *Secale* genus of the Triticeae Dumortier, an important related species of barley and wheat, which is critical for the study of cross-breeding, evolutionary relationship and genetic improvement of Gramineous crops [62]. In this study, we systematically studied the *ScbHLH* family by studying the recently published whole genome sequence of *S. cereale* [63]. We identified 220 *bHLH* genes in *S. cereale*, and their gene structures, motif compositions, repetition events, chromosome distributions, and phylogenetic relationships were observed. These analyses can provide insights into the evolutionary history and functional diversity of the *bHLH* gene family in rye, as well as the potential roles of specific *bHLH* genes in different biological processes. And, the expression patterns of *ScbHLH* members under different stress and hormone induction were further analyzed. Our results provide valuable clues to the functional identification and evolutionary relationship of the *bHLH* gene in *S. cereale*.

## Results

### Identification of *bHLH* genes in *S. cereale*

Two BLAST methods were used to identify all possible *bHLH* genes in the rye genome (Table S1). The resulting genes were subsequently assigned names ranging from *ScbHLH1* to *ScbHLH220* based on their respective positions on the rye chromosomes. A comprehensive analysis was conducted to determine their fundamental characteristics, including gene coding sequence (CDS), protein molecular weight (MW), isoelectric point (PI), and subcellular localization.

Of the 220 ScbHLH proteins, ScbHLH195 was the smallest with 85 amino acids. The largest was ScbHLH82 with 887 amino acids. The molecular weight of the proteins ranged from 9.61 kDa (ScbHLH195) to 96.60 kDa (ScbHLH108). The pI ranged from 4.67 (*ScbHLH31*) to 11.95 (*ScbHLH154*), with a mean of 6.75. All of the ScbHLH proteins contained the HLH domain, which is necessary for their function as transcription factors. Additionally, 15 of the ScbHLH proteins contained the bHLH-MYC domain, which may contribute to additional functions or regulatory mechanisms specific to these genes. In the predicted subcellular localization results, 187 *ScbHLHs* were located in the nucleus, 19 in the chloroplast, 9 in the cytoplasmic, three (*ScbHLH16*, *ScbHLH43*, *ScbHLH168*) in the mitochondria, one (*ScbHLH184*) in the cytoskeleton, one (*ScbHLH220*) in the extracellular compartment, one (*ScbHLH85*) in the peroxisome (Table S1). The ratio of *ScbHLH* genes to total genes in the *S. cereale* genome was approximately 0.50%, which was lower than that in *Arabidopsis* (0.59%) [11] and maize (0.53%) [24], but higher than that in wheat (0.46%) [25], barley (0.35%) [64], rice (0.44%) [7], *S. italica* (0.48%) [33], tomato (0.46%) [19], and buckwheat (0.49%) [31]. This information provides insights into the evolution and diversification of the *bHLH* gene family in different plant species.

### Multiple sequence alignment, phylogenetic analysis, and classification of *ScbHLH* genes

**Fig. 1:**
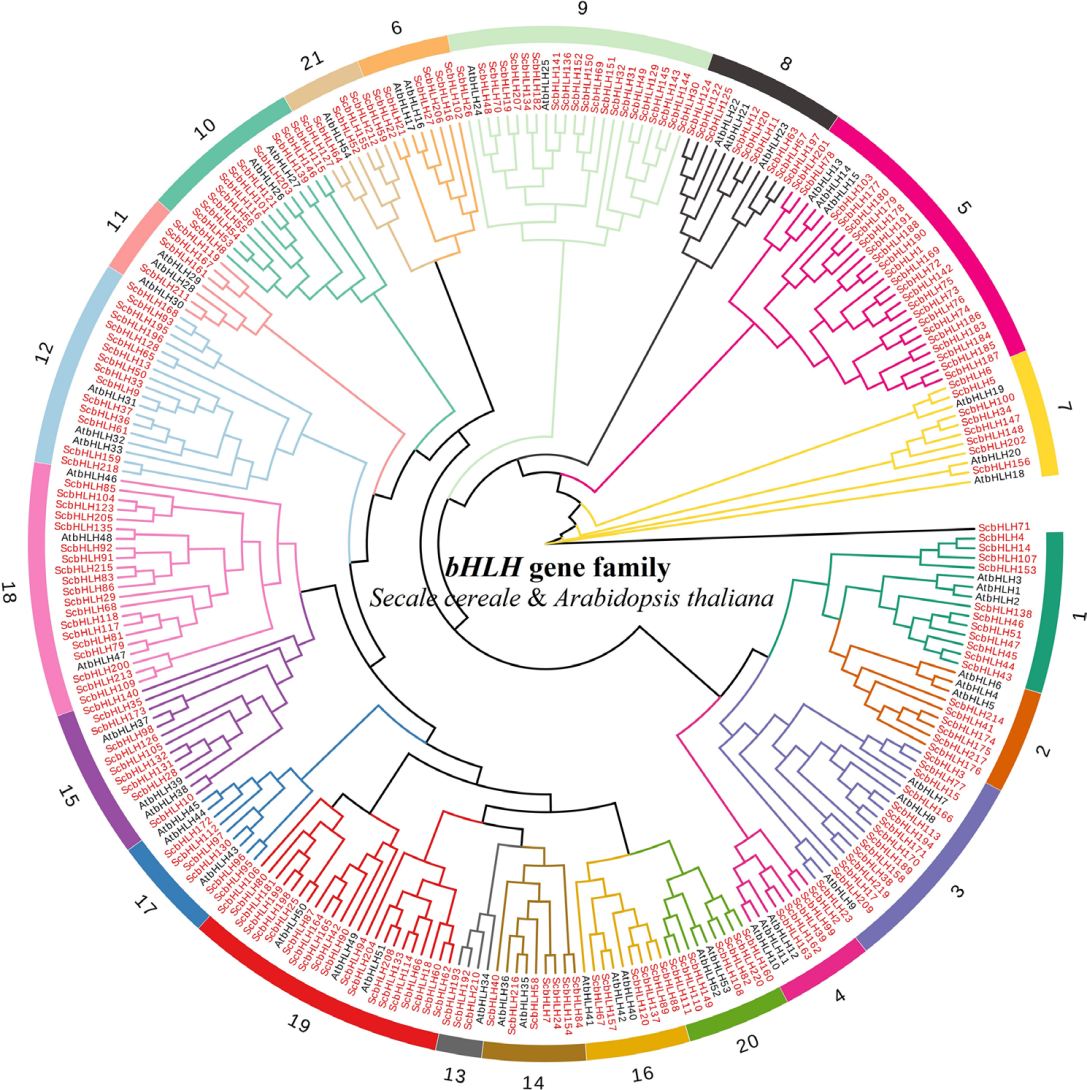
Unrooted phylogenetic tree showing relationships among bHLH proteins of *S. cereale* and *Arabidopsis*. The phylogenetic tree was derived using the neighbor-joining method in MEGA7.0. The tree shows the 21 phylogenetic subfamilies, which are denoted in red font. bHLH proteins from *Arabidopsis* are denoted by the prefix ‘At’

To determine the subfamily classification of bHLH proteins in rye, we constructed phylogenetic trees of *S. cereale* (220 ScbHLHs) and *A. thaliana* (54 AtbHLHs) using the neighbor-joining method (Fig. 1, Table S1). Referring to the classification method proposed by Toledo-Ortiz et al. [11] and Pires and Dolan [17], 274 bHLH proteins are divided into 21 main topological clades (subfamilies 1 ~ 21) and one unclassified protein (ScbHLH71). The 21 main subfamilies contained 219 ScbHLH protein members, which is consistent with the classification of bHLH proteins in *Arabidopsis* [11], indicating that these subfamilies of bHLH proteins have not been lost in *S. cereale* during long-term evolution. The cluster tree shows that ScbHLH71 protein forms a unique topological structure, which is inconsistent with the classification of *Arabidopsis*, it may indicate a new feature of the diversity evolution of *S. cereale* (Fig. 1, Table S1). We have noticed bHLH proteins in wheat [25], barley [64], and other Triticeae Dumortier members, may have generated new evolutionary features that are different from *Arabidopsis* [11], tomato [19], common bean [20], and Moso bamboo [26], but more evidence is needed. Among these subfamilies, subfamily 5 contains the largest number of members (23 ScbHLH proteins), while subfamily 13 has the fewest (three ScbHLH proteins). The topology tree shows a close genetic relationship between some ScbHLH proteins and many AtbHLH proteins (bootstrap support ≥ 70), such as ScbHLH130, ScbHLH216, and ScbHLH98, indicating that these homologous proteins may have similar gene structures and physiological functions.

To discern conserved amino acid residues across various subfamilies, a subset of AtbHLHs and ScbHLHs were randomly selected from 21 distinct subfamilies for comprehensive multi-sequence comparisons (Fig. S1, Table S1). Based on previous studies [11], the basic regions in ScbHLH proteins may be diverse, such as subfamily 7,9,12,13,14, and 20. The amino acid residues in the basic region can regulate the specific binding of bHLHs to DNA [6, 7], which may indicate the complex physiological functions of ScbHLH proteins. As shown in Fig. S1, the bHLH protein domain span of *S. cereale* is about 50 ~ 60 amino acids, which is different from rice [7] and *Arabidopsis* [11].

### Conserved motifs, gene structures, and cis-acting elements analysis of *ScbHLH* genes

**Fig. 2:**
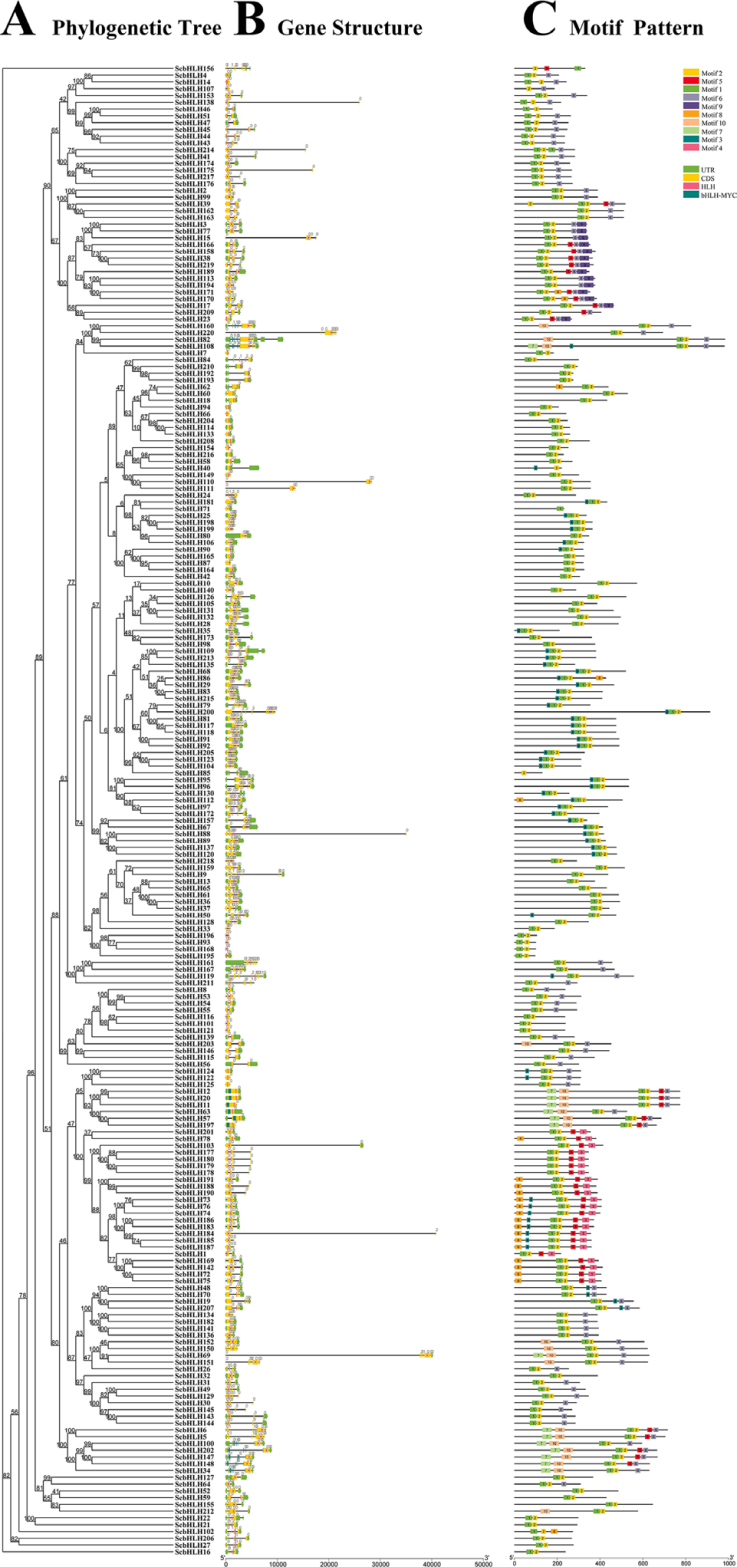
Phylogenetic relationships, gene structure analysis, and motif distributions of *S. cereale* bHLH proteins. (**A**) Phylogenetic tree was constructed using the neighbor-joining method with 1000 replicates for each node. (**B**) Exons and introns are indicated by yellow rectangles and grey lines, respectively. (**C**) Amino acid motifs in the ScbHLH proteins (1–10) are represented by colored boxes. The black lines indicate relative protein lengths

By comparing the DNA structures of these *ScbHLH* members in the rye genome, the number and arrangement characteristics of exons and introns of these genes were further analyzed (Fig. 2, Table S1). The results showed that 220 *ScbHLH* genes had varying number of exons ranging from 1 to 13 (Table S1). Fifteen (6.82%) *ScbHLH* genes contain one exon and are distributed in three different subfamilies (8, 14, and 19). The other genes have two or more exons, with the largest proportion of *ScbHLH* genes (n = 49) having three exons. The member of subfamily 18 (*ScbHLH200*) had the maximum number of exons (13). We note that members of subfamily 11 have similar complex genetic structures, with 9, 11, or 12 exons. Subfamilies 8 and 10 contained one or two exons, except for individual genes (*ScbHLH53*). Subfamilies 9 and 18 have the highest number of exon types, with six different exon types of genes. In general, the gene structures of members of the same subfamily exhibit certain similarities, although their exon and intron distributions may be inconsistent.

Ten conserved motifs were found by the MEME online program in these ScbHLH proteins, and the characteristic amino acid regions of ScbHLH members were arranged (Fig. 2C). The motifs 1 and 2 are widely distributed in most bHLH proteins, except in ScbHLH107, ScbHLH156, ScbHLH40, and ScbHLH71. In most members, the positions of these two motifs are very close. In general, ScbHLH proteins within the same subfamily have similar motif arrangements. For example, subfamilies 2, 3, 4, 8, and 11 contained motifs 1, 2, and 6; subfamily 3 contained motifs 2, 5, and 6; subfamily 5 contained motifs 1, 2, 3, and 5; subfamilies 16 and 17 contained motifs 4, 1, and 2. Almost all members of subfamily 7 contained motifs 7, 10, 1, 2, and 6. In addition, some motifs exist only in specific subfamilies. Motif 3 was specific to subfamilies 5, and 8; motif 9 was specific to subfamilies 3; motif 5 was specific to subfamilies 3, 4, 5, 7, and 8. Further analysis indicates that some motifs are always located at specific locations in the structures of these ScbHLHs. In subfamilies 1, 2, 3, 6, 10, 10, 12, 13, 14, and 15, motifs 1 and 2 are always distributed at the beginning of the pattern. Motif 7 is almost always positioned at the beginning of subfamilies 7 and 8; motif 4 is almost always distributed at the beginning of subfamilies 16, 17, and 18. In subfamily 3, motif 9 is almost always distributed at the end of the pattern. From the arrangement of these motifs, the motif structure of the same subfamily is similar, indicating that the protein structure of the same subgroup may be conservative, which supports the classification of phylogenetic trees.

To analyze the complex physiological processes that these genes may involve, we identified cis-acting elements in the promoter region (upstream 2000 bp) of 220 *ScbHLH* genes. A total of 126 cis-regulating elements have been identified (Table S3), which involve 48 different physiological functions. These cis-regulatory elements could be divided into eight categories: site binding-related, development-related, environmental stress-related, hormone responsive-related, light responsive-related, promoter-related, wound responsive-related, and other elements. Among the promoter elements of the *ScbHLH* genes, light-response elements accounted for the largest proportion, including 36 cis-regulatory factors. Some elements may participate in complex physiological functions, such as the P-box in the *ScbHLH100* promoter, which is a gibberellin-responsive and part of a light-responsive element. Five promoter-related elements (CAAT-box, TATA-box, A-box, Box II-like sequence, and 3-AF3 binding site) in the promoter region were identified in all *ScbHLH* genes. There were 12 hormone-responsive elements in the 213 *ScbHLH* genes of the *S. cereale*, which covered most plant hormones, including abscisic acid-responsive (ABRE, AAGAA-motif), auxin-responsive (AuxRR-core, AuxRE, and TGA-box), gibberellin-responsive (P-box, GARE-motif, and TATC-box), salicylic acid-responsive (TCA-element), and jasmonic acid-responsive elements (TGACG-motif, CGTCA-motif). In addition, cis-regulatory elements related to drought, low-temperature, salt stresses, anaerobic conditions, other defenses, and stress responses were found in *ScbHLH* genes. Nearly 80.45% of *ScbHLH* genes contained abscisic acid-responsiveness and Methyl jasmonate (MeJA) responsive elements; whereas only approximately 49.55% of *bHLH* genes contained gibberellin-responsive elements. Twelve cis-acting elements are involved in the expression of regulatory processes of different tissues (endosperm, meristem, leaf, root, and seed) during development in *S. cereale*. Therefore, the *ScbHLH* genes can not only participate in the development process of multiple tissues but also respond to various abiotic and wound stressed.

### Chromosomal spread and gene duplication of *ScbHLH* genes

**Fig. 3:**
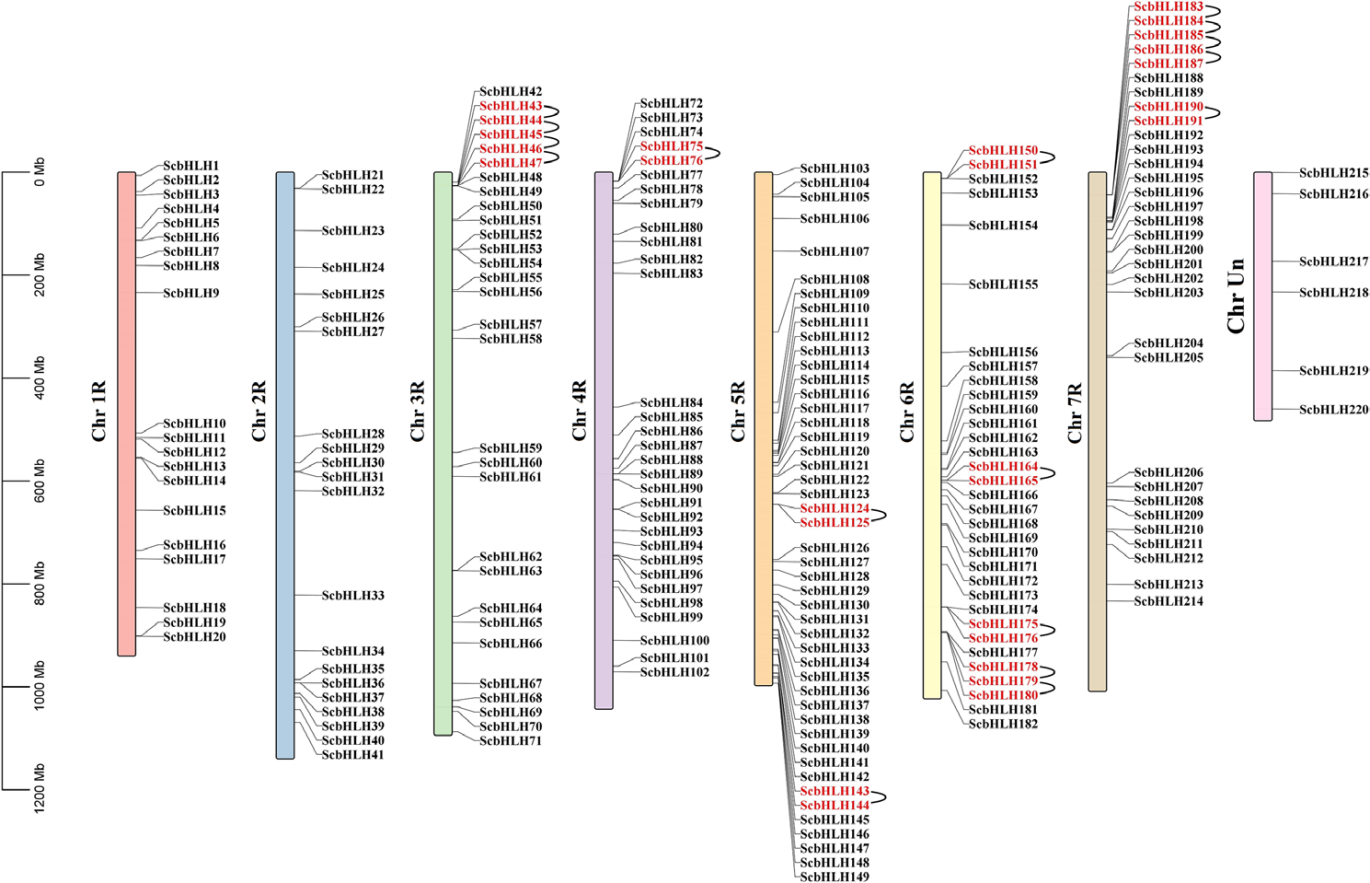
Schematic representation of the chromosomal distribution of the *S. cereale bHLH* genes. Vertical bars represent the chromosomes of *S. cereale*. The chromosome number is indicated to the left of each chromosome. The scale on the left represents chromosome length

**Fig. 4:**
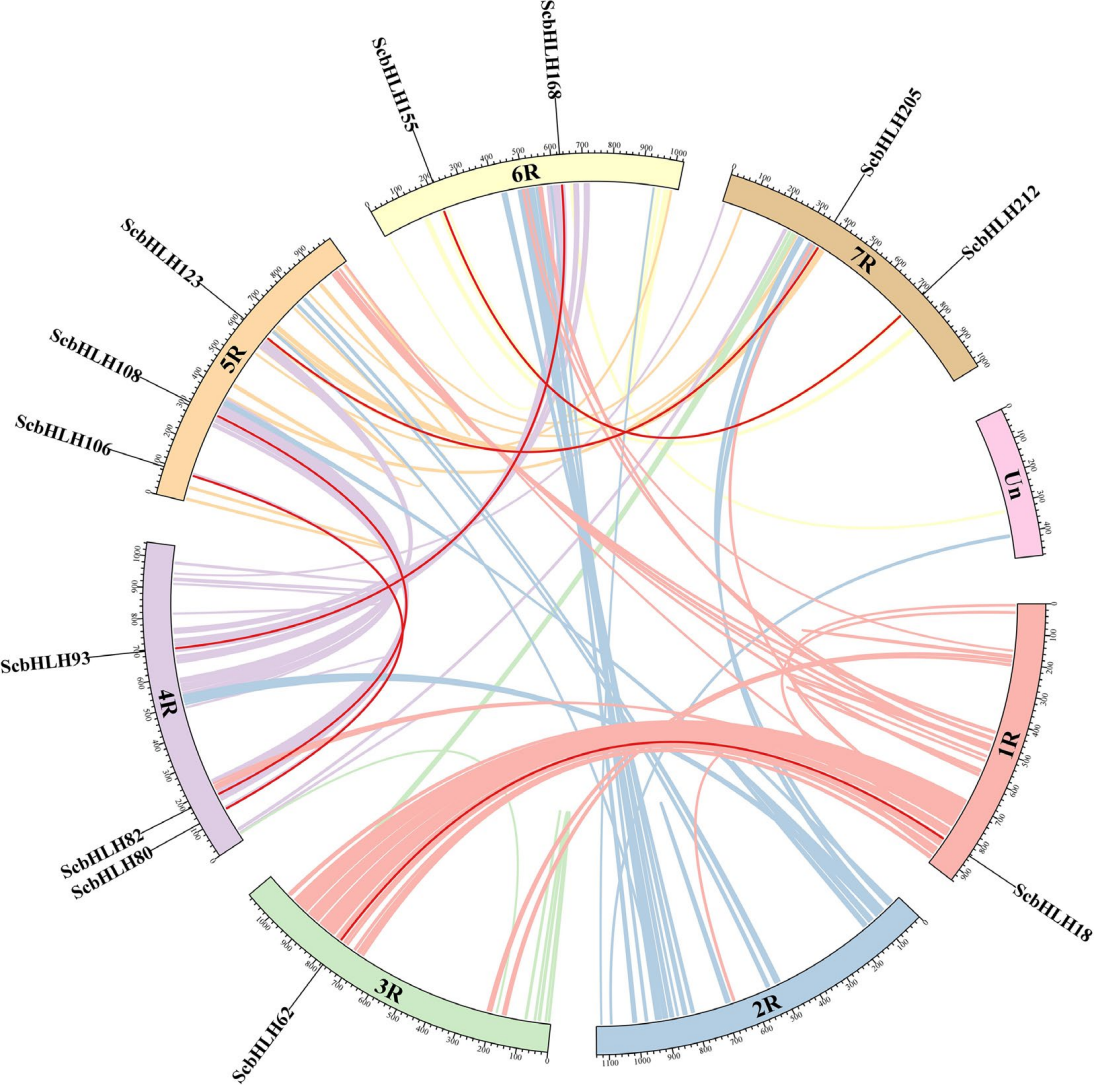
Schematic representation of the chromosomal distribution and interchromosomal relationships of *S. cereale bHLH* genes. Colored lines indicate all synteny blocks in the *S. cereale* genome, and the red lines indicate duplicated *bHLH* gene pairs. The chromosome number is indicated at the bottom of each chromosome

A total of 214 *ScbHLH* genes are unevenly distributed on chromosomes 1R to 7R (Fig. 3, Table S1). To locate all *ScbHLH* members, the unassigned chromosome (ChrUn) based on the annotated sequence was also considered. Moreover, six *ScbHLH* members (*ScbHLH215* to *ScbHLH220*) were located on ChrUn. Each *ScbHLH* gene corresponds to the physical location on different chromosomes in the rye. Among them, chromosome 5R contains the most *ScbHLH* members (47 *bHLH* genes, ~ 21.36%), followed by 6R (n = 33, ~ 15.00%). 1R contained the lowest (n = 20, ~ 9.09%). The chromosomes 7R, 4R, 3R, and 2R contained 32 (~ 14.55%), 31 (~ 14.09%), 30 (~ 13.64%), and 21 (~ 9.55%) *ScbHLH* genes, respectively. The presence of two or more gene members from the same family within the 200 kb chromosome region is defined as the presence of tandem duplications (tandemly arrayed genes) [32, 33]. Some tandem duplications were observed in the *bHLH* family in *S. cereale* (Fig. 3, Table S4). Seventeen tandem duplications involving 27 *ScbHLH* genes were observed on chromosomes 3R, 4R, 5R, 6R, and 7R. All *ScbHLH* genes involved in tandem duplications belong to the same subfamily. Members of subfamily 5 have more tandem duplications, which we speculate may be one of the reasons why this subfamily has the largest number of members. For example, *ScbHLH150* and *ScbHLH151* are tandemly arrayed genes that aggregate in subfamily 9. There were six pairs of segmental duplications (duplications of large chromosomal regions) in *ScbHLH* genes (Fig. 4, Table S5). Chromosomes 4R and 5R had the most *ScbHLH* members (n = 3) and 1R and 3R had the least (n = 1). For all identified *ScbHLH* genes, subfamily 19 had two segmental duplications (four *ScbHLH* genes), while groups 12, 18, 20, and 21 had only one *ScbHLH* gene. These gene replication events formed the expansion of many *ScbHLH* members, which may play an important role in the evolution and the occurrence of new functions in environmental adaptation for the *bHLH* gene family in rye.

### Synteny analysis of *ScbHLH* genes

**Fig. 5:**
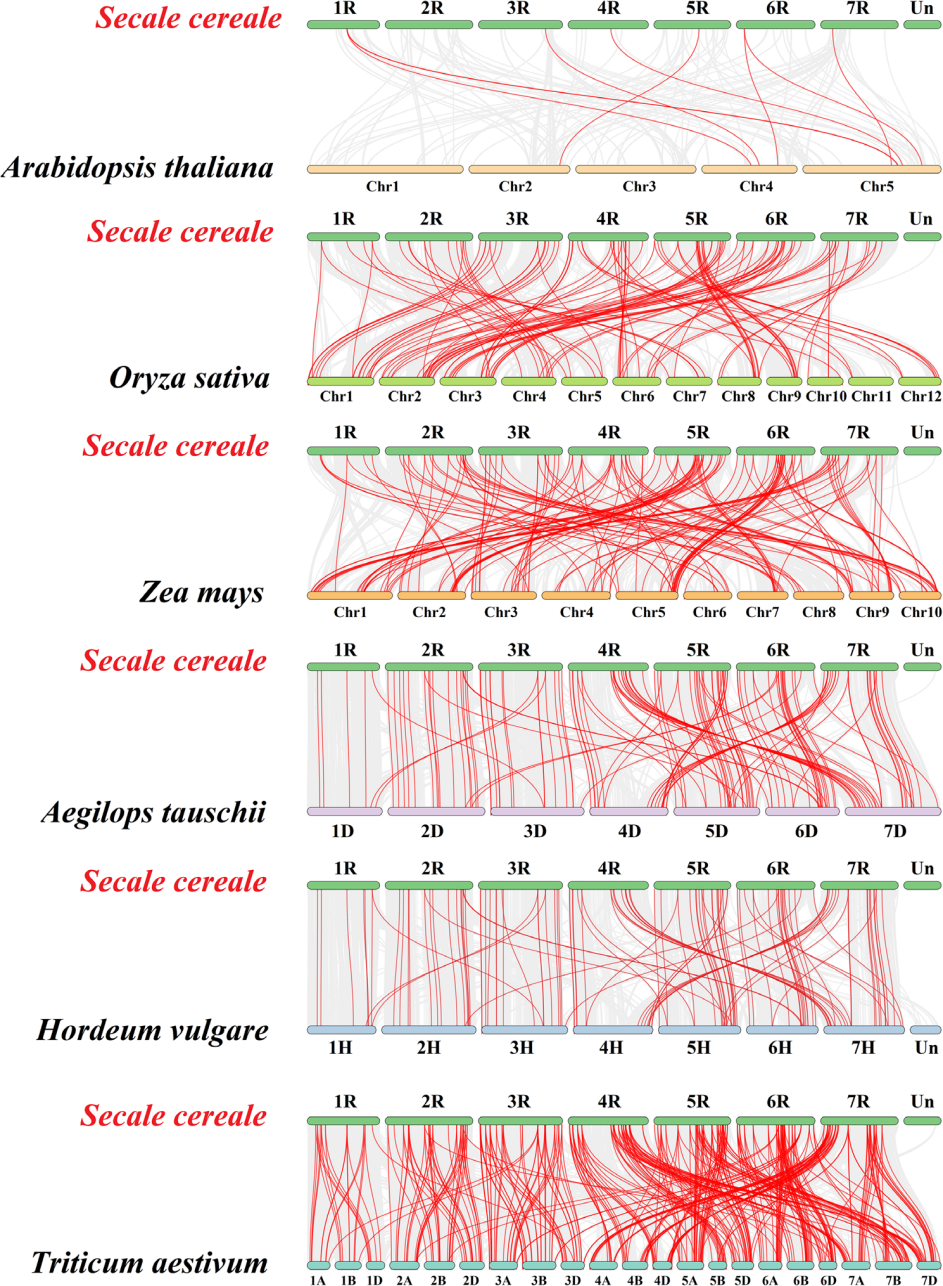
Synteny analyses of the *bHLH* genes between *S. cereale* and six representative species (*T. aestivum*, *A. tauschii*, *H. vulgare*, *O. sativa*, *Z. mays*, and *A. thaliana*). Gray lines on the background indicate the collinear blocks in *S. cereale* and other plant genomes; red lines highlight the syntenic *S. cereale bHLH* gene pairs

To elucidate the evolutionary relationships between these bHLH proteins in several plants, the synteny maps based on homologous genes from rye and six representative plants were constructed. Five monocotyledonous plants included three Triticeae Dumortier plants (*Triticum aestivum*, *Aegilops tauschii*, *Hordeum vulgare*), one model plant (*Oryza sativa*), and one C4 plant (*Zea mays*). In addition, a dicotyledonous model plant (*A. thaliana*) was also included in the comparison (Fig. 5, Table S6). 148 ScbHLH proteins exhibit homologous relationships with those in *A. thaliana* (7 genes), *O. sativa* (88), *Z. mays* (88), *A. tauschii* (112), *T. aestivum* (138), and *H. vulgare* (100) (Table S6). The number of collinear gene pairs between rye and other representative species (*A. thaliana*, *O. sativa*, *Z. mays*, *A. tauschii*, *T. aestivum*, and *H. vulgare*) was 9, 116, 147, 133, 380, and 117, respectively. In the orthologous pairs of the Triticeae Dumortier, rye with *A. tauschii* and *H. vulgare* involved a relatively high proportion of *bHLH* genes, which was 85.47% and 84.21%, respectively. Ten *ScbHLHs* paired with at least two genes in five monocots, notably including *S. cereale* and other members of the Triticeae Dumortier plant family. These genes included *ScbHLH28*, *ScbHLH62*, *ScbHLH82*, *ScbHLH86*, *ScbHLH108*, *ScbHLH123*, *ScbHLH155*, *ScbHLH159*, *ScbHLH205*, and *ScbHLH212*, indicated that they may have an undeniable role in the evolution of these monocotyledonous plants.

Moreover, a considerable number of collinear gene pairs (consisting of fifty *ScbHLH* genes) were discovered between *S. cereale* and Triticeae Dumortier plants, with genes that were not found in *A. thaliana*, *O. sativa*, and *Z. mays*. These included *ScbHLH5* with *AET1Gv20229700* / *ARI1A01G110900* / *HORVU1Hr1G020370* and *ScbHLH65* with *AET1Gv20762600* / *ARI3A01G346700* / *HORVU1Hr1G066250*, indicating that these *ScbHLH* genes may exhibit stronger expansion within the Triticeae Dumortier plants, which may be different from dicotyledonous plants such as *Arabidopsis*, and other monocotyledonous plants. Furthermore, in these dicotyledonous and monocotyledonous plants, many *ScbHLH* genes have been found to have at least one pair of homologous pairs (especially between *S.cereale* and *H.vulgare*). For example, *ScbHLH11*, *ScbHLH65*, *ScbHLH86*, *ScbHLH120*, *ScbHLH154*, and *ScbHLH198*, indicated that these homologous genes may have been preserved in long-term evolution, and may exist before the differentiation of ancestral plants. To better distinguish the targeted or balanced selection of these 220 *ScbHLH* genes, these members were subjected to Tajima-D neutrality testing (Table S7). The D value is 13.82 (significant deviation from 0), which indicates that the *ScbHLH* gene family may participate in the evolution of neutral selection.

### Evolutionary analysis of *ScbHLH* and *bHLH* genes of several different species

**Fig. 6:**
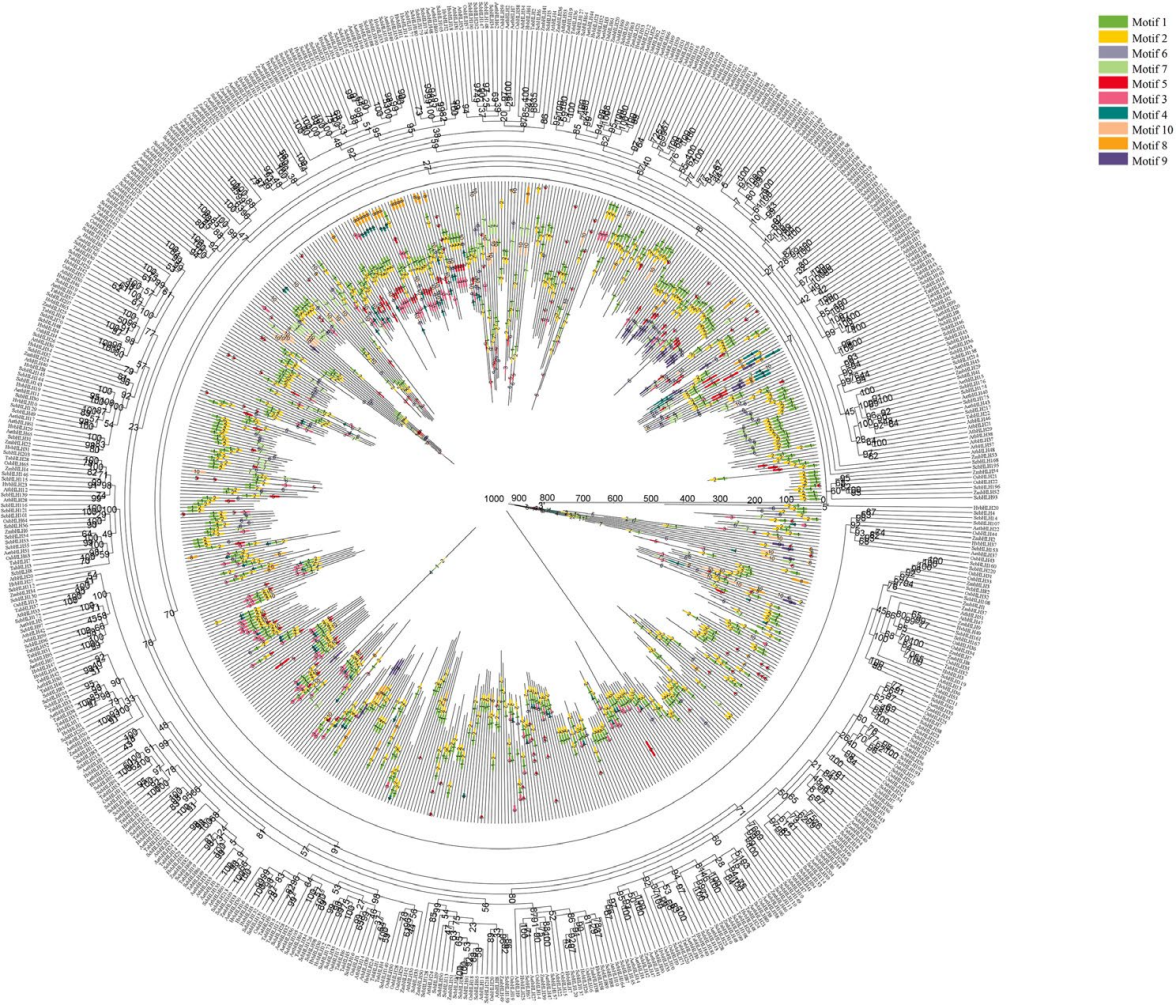
Phylogenetic relationship and motif composition of the bHLH proteins from *S. cereale* with six different plant species (*T. aestivum*, *A. tauschii*, *H. vulgare*, *O. sativa* subsp. *Indica*, *Z. mays*, and *A. thaliana*). Outer panel: an unrooted phylogenetic tree constructed using Geneious R11 with the neighbor-joining method. Inner panel: distribution of conserved motifs in bHLH proteins. The differently colored boxes represent different motifs and their positions in each bHLH protein sequence. The sequence information for each motif is provided in Table S2

To analyze the genetic relationship of the bHLH proteins between rye and these six representative plants (*A. thaliana*, *O. sativa*, *Z. mays*, *A. tauschii*, *T. aestivum*, and *H. vulgare*), an unrooted Neighbor-joining tree was constructed. Based on the MEME online service software, 10 conserved motifs were identified in the sequence of 589 bHLH proteins from these plants (Fig. 6, Table S2). The detailed conservative motif domains are shown in Tables S1 and S2. ScbHLH proteins tend to aggregate with the bHLH members of *A. tauschii*, *T. aestivum*, and *H. vulgare*. Except for a few ScbHLH proteins, such as ScbHLH71, ScbHLH85, and ScbHLH107, all other ScbHLH proteins contained motifs 1 and 2. Moreover, some motifs only exist in specific topological structures, such as motifs 7, 8, and 10. Overall, these bHLH genes of Triticeae Dumortier plants and *S. cereale* on the same topological branches had similar motif arrangements. In these plants, some specific bHLH protein subfamilies often contain similar motifs, which suggests their evolutionary relationship. Motifs 1, 2, 5, and 3 form a conserved structure and tend to aggregate within subfamily 5, while motifs 7, 10, 1, 2, 5, and 6 tend to aggregate within subfamily 7.

### Expression patterns of *ScbHLHs* in several plant organs

**Fig. 7:**
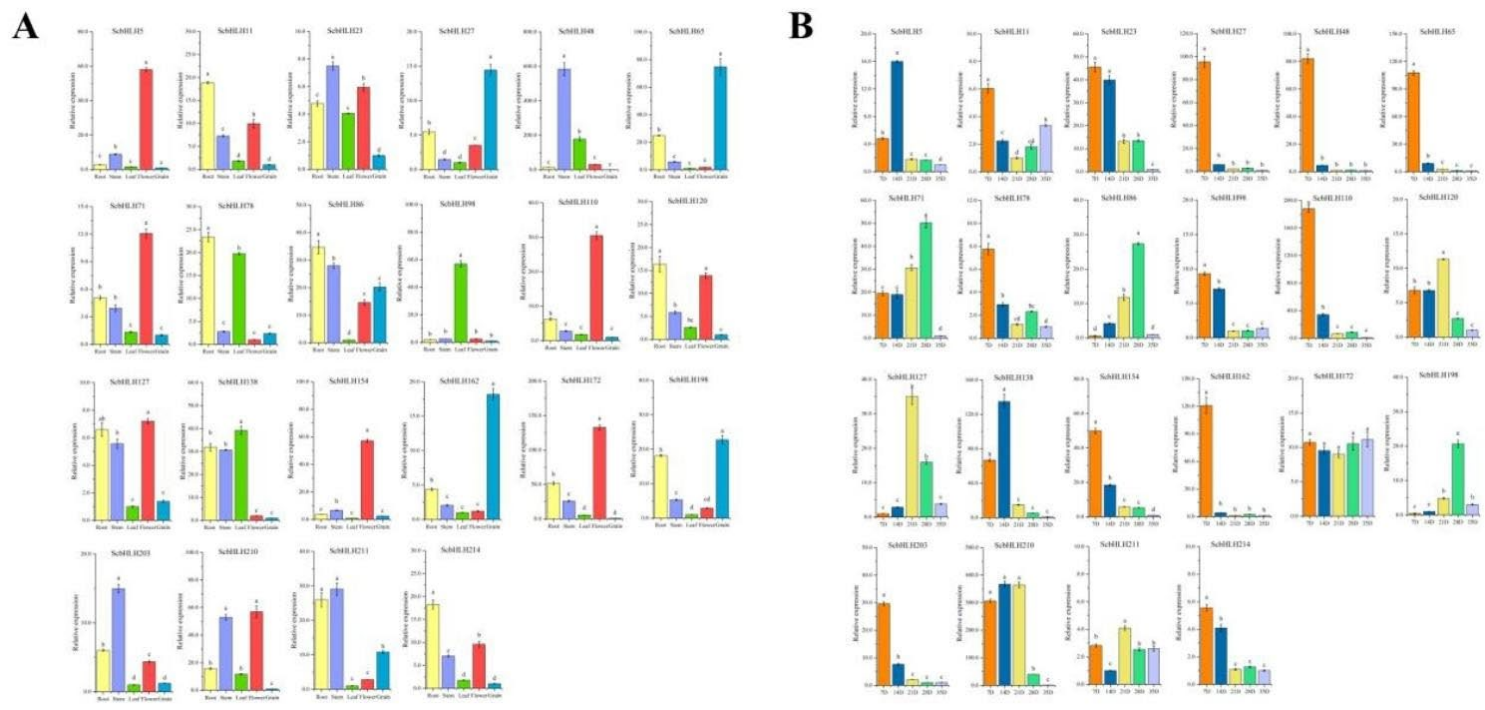
Expression patterns of 22 *S. cereale bHLH* genes in several plant organs. (**A**) Expression patterns of 22 *S. cereale bHLH* genes in the roots, stems, leaves, flowers and grains were examined via qRT-PCR. Error bars were obtained from three measurements. Lowercase letters above the bars indicate significant differences (α = 0.05, The least significant difference test [LSD]) among the treatments. (**B**) Expression patterns of 22 *S. cereale bHLH* genes were examined during different grain development stages: 7 DPA (early filling stage), 14 DPA (mid filling stage), 21 DPA (early ripening stage), 28 DPA (mid ripening stage), and 35 DPA (full ripening stage). The statistical method is consistent with Fig. 7A

To evaluate the physiological functions of 22 *bHLH* genes in *S. cereale*, we used qRT-PCR to detect the expression levels of 22 Sc*bHLH* members. As far as possible, the selected members of different subfamilies exhibit significant differences in amino acid structures and distant clustering relationships. The accumulation of transcripts of these *ScbHLH* genes in five organs (leaf, stem, root, flower, and grain) was detected (Fig. 7A). Many *ScbHLH* genes are preferentially expressed in certain tissues of *S. cereale*. Eight genes (*ScbHLH11*, *ScbHLH78*, *ScbHLH86*, *ScbHLH120*, *ScbHLH138*, *ScbHLH198*, *ScbHLH211*, and *ScbHLH214*) displayed the highest expression in the root; the expression of six genes (*ScbHLH23*, *ScbHLH48*, *ScbHLH138*, *ScbHLH203*, *ScbHLH210*, and *ScbHLH211*) was highest in the stem, whereas the expression of *ScbHLH98* and *ScbHLH138* was highest in the leaf, and the expression of seven genes (*ScbHLH5*, *ScbHLH71*, *ScbHLH110*, *ScbHLH120*, *ScbHLH154*, *ScbHLH172*, and *ScbHLH210*) was highest in the flower, and *ScbHLH27*, *ScbHLH65*, *ScbHLH162*, and *ScbHLH198* were highly expressed in the grain.

Some *ScbHLH*s may regulate the grain development of *S. cereale*, thus affecting its nutritional composition and development rate. To determine the genes that may regulate the development of rye grain, the expression of 22 *ScbHLH* genes from five grain-filling stages after flowering were evaluated. The expression levels of most *ScbHLH* genes were different in grain during the five grain development stages. In the grain of *S. cereale*, the expression of most genes decreased with grain development. The expression levels of four genes (*ScbHLH11*, *ScbHLH23*, *ScbHLH138*, and *ScbHLH210*) were highest at 14 DPA(days post-anthesis), whereas the expression levels of three genes (*ScbHLH71*, *ScbHLH86*, and *ScbHLH198*) were highest at 28 DPA (Fig. 7B). The expression levels of most *ScbHLH* genes, except for *ScbHLH11*, and *ScbHLH172*, were lowest at the full-ripening stage.

In addition, the expression patterns of some *ScbHLH* members were observed to exhibit some possible coordinated expression in several plant organs (Figs. S2 and S3). Most *bHLH* members exhibit a significant positive correlation. For example, nine genes *ScbHLH5*, *ScbHLH23*, *ScbHLH71*, *ScbHLH110*, *ScbHLH120*, *ScbHLH127*, *ScbHLH154*, *ScbHLH172*, and *ScbHLH210* were significantly positively correlated in several plant organs, and *ScbHLH11*, *ScbHLH23*, *ScbHLH27*, *ScbHLH48*, *ScbHLH65*, *ScbHLH78*, *ScbHLH98*, *ScbHLH110*, and *ScbHLH154* were significantly positively correlated the five development in grain. And, some pairs of *ScbHLH* genes (*ScbHLH98* and *ScbHLH86* / *ScbHLH127*) showed a significant negative correlation.

### Expression patterns of *ScbHLH* genes in response to different treatments

To further observe if the transcription patterns of these rye *bHLH* members may change under different abiotic stress, the rye seedlings were examined under six abiotic stresses, including ultraviolet radiation (UV), flooding, PEG (10%), NaCl (5%), heat (40˚C), and cold (4˚C). Based on qRT-PCR, the expression patterns of 22 *ScbHLH* genes responding to different abiotic stress in roots, leaves, and stems were analyzed (Fig. S4). Many *ScbHLH* members are significantly up-regulated or inhibited under different stresses. Most *bHLH* genes are sensitive to environmental changes, and their expression levels show significant differences under short-term stress, indicating that they may be involved in these stress regulations. For example, the expression of *ScbHLH11*, *ScbHLH27*, and *ScbHLH86* significantly increased in roots, stems, and leaves in 1 h during heat stress. In addition, with the increase in treatment time, the expression level of *ScbHLH* showed changes in different organs, depending on the specific treatment. The *ScbHLH86*, *ScbHLH211*, and *ScbHLH214* were significantly up-regulated and then down-regulated under cold stress. *ScbHLH48* expression was gradually significantly up-regulated in the roots, whereas it was significantly down-regulated in the stems. Interestingly, many *ScbHLH* genes exhibit opposite expression patterns in these abiotic stresses. For example, the transcript levels of *ScbHLH5* were up-regulated in cold, but down-regulated in ultraviolet radiation treatment in leaves. The expression of many *ScbHLH* genes showed similar patterns during these stress treatments. For example, *ScbHLH86* and *ScbHLH154* expression was significantly up-regulated first and then down-regulated in the leaves by many different treatments. Other genes exhibit characteristics in specific tissues and exposure times. For example, *ScbHLH110* responded quickly to flooding treatment in roots and stems, while *ScbHLH198* showed a significant response to cold treatment in leaves. Negative correlations were observed between most *ScbHLH* genes (Fig. S5). However, some *ScbHLHs* are significantly positively correlated, such as *ScbHLH23*, *ScbHLH71*, *ScbHLH138*, *ScbHLH154*, as well as *ScbHLH5*, *ScbHLH110*, and *ScbHLH210* (P < 0.05).

The *bHLH* family of rye is speculated to be involved in abiotic stresses and hormone responses. Therefore, we considered detecting the expression patterns of these members in the grain-filling period under different hormone treatments. Three representative hormones were considered for application, namely gibberellin, auxin, and abscisic acid. The expression levels of these members were used to construct a correlation network (Fig. S6). Overall, the expression of some genes may be synergistic. The expression of these genes, *ScbHLH5*, *ScbHLH98*, *ScbHLH154*, and *ScbHLH162*, were positively co-expressed under gibberellin induction, and *ScbHLH23*, *ScbHLH48*, *ScbHLH172*, and *ScbHLH211*, were positively co-expressed under auxin induction. In addition, the expression patterns of some genes may be contradictory, for example, the expression pattern of *ScbHLH154* is significantly positively correlated with *ScbHLH48*, *ScbHLH138*, *ScbHLH172*, and *ScbHLH210*, while it is negatively correlated with *ScbHLH65*, *ScbHLH110*, and *ScbHLH162* under short-term treatment with abscisic acid. This reflects the complexity of the functions in different subfamily members, which is speculated to be related to the diversity of promoter elements and amino acid structure. However, more evidence is needed to illustrate it.

## Discussion

### *ScbHLH* gene structures and evolutionary analyses

In this study, a total of 220 *ScbHLH* genes were discerned, each encoding proteins that displayed noteworthy structural variations, thus elucidating the profound intricacies in the *S. cereale*. (Figures 1 and 2, Table S1). The N-terminal base region of the bHLH transcription factor was considered to have a conserved amino acid region that determines DNA binding ability. In general, transcription factors with similar gene structures bind to the specific cis-acting elements [10, 11]. A total of 163 ScbHLHs (74.1%) were classified as E-box binding proteins, 121 ScbHLHs (55.0%) were classified as G-box binding proteins, and 42 ScbHLHs (19.1%) were classified as non-G-box binding proteins. Due to the lack of Glu-13 or Arg-16 in the basic region in the alkaline region, the remaining 18 bHLH proteins (8.2%) are considered to have lost their ability to bind to DNA (Fig. S1, Table S1). This ratio may be similar in monocotyledonous plants, especially in the Triticeae Dumortier plants [25, 32, 33, 65]. Although most ScbHLH proteins have G-box binding ability, their relative abundance is lower than those of *S. italica* (n = 99, 56.9%), and *O. sativa* (n = 95, 56.9%) [12, 33]. Based on the conserved amino acid structures of bHLH proteins, they can function by forming homologous and heterologous complexes [66]. However, the specific molecular mechanisms have not yet been fully understood. Previous studies have reported that the HLH domain has the function of forming dimers, and the Leu-23 and Leu-52 amino acid residues located in the helixes regions are essential for forming dimers [12].

According to the phylogenetic tree constructed by Li et al. [7], similar to *Arabidopsis* [11], rice [7], millet [33], wheat [25], and barley [64], these 21 subfamily members have been preserved. The diversity of bHLH proteins may already exist in early terrestrial plants, possibly earlier than before the isolation of monocotyledonous and dicotyledonous plants. In addition, an unclassified gene (UC, *ScbHLH71*) is independently supported in the topological structures of bHLH proteins, which is speculated to be further differentiation of the *bHLH* genes in *S. cereale*, which has also been observed in some monocotyledonous plants such as sorghum [32] and barley [64]. Although some proteins may have more intense evolution or expansion, the distance within different families is convergent, that is, the bHLH proteins may have a closer genetic distance in the same family of plants, whether C3 or C4 plants. These bHLH proteins in Gramineae plants may come from one or several ancestral proteins, which is supported by the distant genetic relationship between angiosperms and animal bHLH proteins [10]. Based on the topology tree analysis, it was observed that the most numerous rye subfamilies were: 5 (n = 23, 10.45%), 9 (n = 21, 9.55%), 19 (n = 20, 9.09%) and 18 (n = 19, 8.64%). This pattern differs from *Arabidopsis* but aligns with findings in sorghum [32] and barley [64]. The similarity in member count suggests that these specific subfamilies of *bHLH* genes have experienced extensive expansion during the long evolutionary process of monocotyledonous plants. The number and proportion of members in subfamilies 1, 5, and 9 were significantly higher than those in *Arabidopsis* [11], and similar to *S. italica* [33]. Whether this differentiation is beneficial for Gramineae or Triticeae Dumortier plants has not yet been determined.

Most *ScbHLH* genes (n = 205, 93.18%) contain more than two exons. Some subfamilies have observed the complexity of the number of introns, mainly reflected in subfamilies 9, 11, 18, and 19, which may lead to the expansion of the *bHLH* family and the generation of new functions in *S. cereale*. In addition, previous studies have shown that genes with few or no introns are usually less expressed in plants [67]. The non-intron genes mainly belong to subfamily 8, which is consistent with sorghum [32] and millet [33]. Tandem duplications can rapidly expand and contract in response to environmental changes, maintaining an equal number of functionally related genes, without increasing genetic complexity during evolution [68]. In addition, segmental duplications are commonly present in some animal and plant genomes, which helps to form genetic diversity [69]. Therefore, tandem and segmental duplications contribute to the expansion of this gene family and play an important role in genome evolution. As expected, all amplified events are within this subfamily, including tandem and segment duplications, similar to *Arabidopsis* [11], rice [7], and buckwheat [31], indicating that repeated events did not cause gene expansion between different subfamilies. However, tandem duplications of the *ScbHLH* genes may have a higher contribution to the amplification of the *bHLH* family in *S. cereale*, which is inconsistent with *S. bicolor* [32] and *S. italica* [33]. These gene replication events formed the expansion of many *ScbHLH* members, which may play an important role in the evolution and the occurrence of new functions in environmental adaptation for the *bHLH* gene family in rye.

### Expression patterns and function prediction of *ScbHLHs*

In this investigation, the expression patterns of 22 *ScbHLH* genes exhibiting significant distinctions in phylogenetic trees were analyzed across various organs and developmental stages of grain (Fig. 7). Almost all *ScbHLH* genes were found to exhibit significant differential expression (more than 2-fold differences). *ScbHLH86* is classified to subfamily 18, which has the highest expression in stems, roots, flowers, and grains. Similar to the expression pattern of the homologous gene *CIB2* (*AT5G48560*) in *Arabidopsis*, which participates in roots and stems development and activates FT protein regulation of flowering and grain-filling processes by forming various combinations of heterodimers with CIB4, CIB5, and CIB1. *ScbHLH120*, classified in subfamily 16, is highly expressed in the flowers and roots in *S. cereale*, and its homologous gene *HBP1* (*Os08g0506700*) interacts with POH1 to directly regulate the expression of the key factor *Hd1* during rice flowering [70]. In addition, the expression pattern of *ScbHLH120* was observed to be similar to the homologous gene (*AT4G30410*) in *Arabidopsis* [71]. *ScbHLH78* showed the highest expression in roots and leaves, which is consistent with the expression pattern of the same family gene *AT2G2270*. *AT2G22770*, belonging to the subfamily 5, may play a key role in the roots and leaves of *Arabidopsis* [72]. The expression of *AT1G06170*, a member of the subfamily 4, is higher in grains than in roots, stems, leaves, and flowers [73], similar to the expression pattern of *ScbHLH162*. The expression of most *ScbHLH* genes was significantly positively correlated in *S. cereale*, indicating that their combination may have synergistic effects in five plant organs (Fig. S2). These findings provide direction for the study of their gene function. As a Triticeae Dumortier plant, the grain development process is very important for *S. cereale*, which is divided into five representative stages. Furthermore, the *ScbHLH* genes that may regulate the development of rye grains have been identified. This study investigated the expression levels of 22 *bHLH* members in grains during the filling period. The results showed that most of these genes were expressed in the early and middle stages of grain filling. *ScbHLH172* is stably expressed at almost all stages. In addition, *ScbHLH71* and *ScbHLH210* have the highest expression in the early and middle stages of grain filling. We attempt to further validate the role of these genes in the growth and grain development of *S. cereale*.

The growth and developmental processes in plants are intricately influenced by both external environmental factors and hormonal cues. Rye, as a cold and drought-tolerant crop, exhibits the ability to adapt to stress conditions and respond to hormone stimulation through elaborate endogenous networks and transcriptional signals, akin to its counterparts wheat and barley [74, 75]. However, a comprehensive understanding of its response to complex abiotic stresses remains elusive. In our study, we conducted a systematic analysis of the expression patterns of 22 *ScbHLHs* in rye seedlings subjected to various stresses (Fig. S4), encompassing six distinct abiotic stresses. Under drought stress conditions, we observed a significant upregulation in the expression levels of 18 *ScbHLH* genes in the roots, 21 genes in the leaves, and 14 genes in the stems, with variations depending on the duration of treatment. These molecular responses likely contribute to the adaptive mechanisms of *S. cereale* in arid environments, aligning with its characterization as a drought-tolerant crop. Different *bHLH* subfamilies may play complex physiological roles in the environmental adaptation process of rye. In *Arabidopsis*, *AT1G63650* (*AtbHLH18*), belonging to subfamily 7, displays preferential expression within *Arabidopsis* root epidermal cells and is speculated to play a role in stress adaptation through the regulation of root development [76]. *ScbHLH5*, member of subfamily 7, displayed remarkable responses at the roots in multiple abiotic stresses. Within the subfamily 8, *ZBF1* (*At1g32640*) encodes an MYC-bHLH-related transcriptional activator that plays a vital role in drought stress and ABA response [77, 78]. Similarly, the expression of *ScbHLH11* was significantly upregulated in almost all abiotic stresses, which may enhance the adaptability of rye to the environment in a similar manner. The expression of some members, such as *ScbHLH23*, *ScbHLH86*, and *ScbHLH214*, significantly up-regulated under flooding stress, which may contribute to stomatal expansion and increased respiration in leaves. The correlation network indicates that these *bHLH* transcription factors are involved in a complex cross-regulatory network regarding stresses and hormone induction. For example, *ScbHLH5* and *ScbHLH65* exhibit a significant positive correlation under different hormone induction, indicating that they have synergistic regulatory effects under various endogenous metabolism.

## Conclusion

In summary, we provided a systematic genome-wide analysis of the *bHLH* gene family in *S. cereale.* These 220 members were divided into 21 subfamilies and an unclassified gene, and their evolutionary relationships, gene structures, conserved motifs, gene replications, and expression patterns were further analyzed. Both tandem duplications and segmental duplications contribute to the expansion of the *bHLH* family in *S. cereale*. Tandem duplications may play a crucial role in the expansion of the *bHLH* genes in the evolution of rye. In addition, the expression patterns of *ScbHLH* in different tissues and grain-filling stages were observed, as well as their responses to different stresses and hormones, were detected. Some candidate genes related to tissue development and environmental stresses have been screened, such as *ScbHLH11*, *ScbHLH48*, and *ScbHLH172*. These data will provide a useful aid for future research on the function of the *bHLH* gene family in rye.

## Materials and methods

### Gene identification

We downloaded the latest high-quality reference genome of *S. cereale* from the NCBI (National Center for Biotechnology Information) GenBank website, the access number is JADQCU000000000 [79]. Firstly, all of the bHLH proteins of *Arabidopsis* (54 AtbHLHs) [11] were used to search for candidate bHLH proteins from the rye genome via the BLASTp program [80]. The candidate genes were searched by BLASTP using a score value of ≥ 100 and an e-value ≤ e − 10. Secondly, the Hidden Markov Model (HMM) file of the bHLH domain (PF00010) is downloaded from the PFAM protein family database (http://pfam.xfam.org/). Based on the HMM model in the HMMER3.0 online software, the bHLH protein sequence in *S. cereale* was identified with a decision value of 0.01 (http://plants.ensembl.org/hmmer/index.html) [81]. Based on PFAM and SMART in thread sequencing, conserved motifs were found in the bHLH proteins in rye (http://smart.embl-heidelberg.de/) [82, 83]. Accordingly, a total of 220 ScbHLH proteins were identified in the rye. Then, in the NCBI protein database, these ScbHLH proteins were used as the initial query for re-verification (https://blast.ncbi.nlm.nih.gov/Blast.cgi? PROGRAM = blastp&PAGE_TYPE = BlastSearch&LINK_LOC = blast home). Finally, the ExPasy online program was used to identify the basic features of the *bHLH* gene in *S. cereale*, including sequence length, protein molecular weight, isoelectric points, and subcellular localization (http://web.expasy.org/protparam/).

### *bHLH* gene structures and conserved motif analysis

Based on the default parameters of the ClusterW program, 220 rye bHLH proteins were subjected to multiple sequence alignment projects to further analyze their characteristic domains [84]. MEGA 7.0 and GeneDoc 2.7(http://genedoc.software.informer.com/2.7/) were used for the manual regulation of conserved domains in the amino acid sequences of these bHLH proteins. Gene Structure Display Server (GSDS; http://gsds.cbi.pku.edu.cn) online software was used to analyze the exon-intron Substructure of these *ScbHLH* genes [85]. Then, the MEME online program (http://meme.nbcr.net/meme/intro. HTML) was used to analyze the conserved motifs and gene structural differences in these bHLH proteins [86]. The optimization parameters for conservative motifs were as follows: the maximum number was 10, and the optimal width of residues was 6 to 200 [32]. In addition, The PlantCARE online software was used to predict cis-acting elements in the upstream 2000 bp promoter region of 220 *bHLH* genes (http://bioinformatics.psb.ugent.be/webtools/plantcare/html/?tdsourcetag=s_pcqq_aiomsg) [87].

### Chromosomal distribution and gene duplication

Firstly, based on the physical location of these genes in the annotation file, all *ScbHLH* genes have been designated as chromosomal details. And, Circos software was used to analyze these *ScbHLH* genes for chromosomal location information [88]. Multiple collinear scanning toolkits (MCScanX) were used to analyze gene replication events based on default parameters [81, 89]. The homology of the *bHLH* genes between *S. cereale* and six other plants (*T. aestivum*, *A. tauschii*, *H. vulgare*, *O. sativa* subsp. *Indica*, *Z. mays*, and *A. thaliana*) was analyzed by using the project of dual synteny plotter in TBtools software (v1.120) [90].

### Phylogenetic analysis and classification of the *ScbHLH* family

According to the classification of AtbHLH proteins, 220 bHLH proteins in *S. cereale* are divided into 21 main subfamilies. In MEGA 7.0, the Jukes-Cantor model is used to construct NJ (neighbor-joining method) trees. The bootstrap value of the constructed phylogenetic tree was set to 1000, and assigned with Geneious R11 with BLOSUM62 cost matrix. In addition, this study also constructed a multi-species phylogenetic tree containing bHLH protein sequences from rye and six plant species (*T. aestivum*, *A. tauschii*, *H. vulgare*, *O. sativa* subsp. *Indica*, *Z. mays*, and *A. thaliana*), which were obtained from the UniProt website [32, 33].

### Plant materials, growth conditions, and abiotic stresses in *S. cereale*

The study employed *S. cereale* cv. Weining, is a well-established traditional variety native to Guizhou Province in southwest China. Since 2022, ‘Weining’ has been cultivated within the greenhouse facilities at Chengdu University Farm. The seedling tray was put in the greenhouse. Rye cultivation entailed potting the plants in a blend of soil and vermiculite (in a 1:1 ratio), within a controlled growth chamber. The chamber was regulated to maintain a diurnal cycle of 16 h at a temperature of 25℃, followed by 8 h at a temperature of 20℃ during the night, while sustaining a relative humidity level of 75%. Plant nutrient solution (10 g of urea, 6 g of potassium dihydrogen phosphate, 2 g of calcium sulfate, and 1 g of magnesium sulfate dissolved in 20 l) was sprayed once a month, with 300mL sprayed into potted plants each time. Generally, pure water irrigation was carried out every 5 days. At the early ripening stage of rye, we carefully collected samples from the roots, stems, leaves, flowers, and grains. Due to the inconsistent filling period of all spikelets in rye, individual spikelets were peeled off with tweezers. Spikelets in the flowering stage (prior to filling stage), including the florets with outer lemma, inner lemma, stamens and pistils were called flowers. For grain analysis, we focused on five key developmental stages: 7 days (early filling stage), 14 days (mid-filling stage), 21 days (early ripening stage), 28 days (mid-ripening stage), and 35 days (full ripening stage). Each organ sample was obtained from five individual plants grown under identical growth conditions. To preserve their physiological state, the samples were swiftly placed in liquid nitrogen, pre-cooled to maintain their integrity, and subsequently stored at -80℃ until further use. Total RNA was rapidly extracted from these samples for subsequent qRT-PCR analysis, with each sample being subjected to at least three technical replicates. Furthermore, we also investigated the expression patterns of these *ScbHLH* genes in rye seedlings subjected to six different abiotic stresses. Rye seeds were sown in seedling trays, and after 28 days of germination, the seedling trays were treated with a 50 mL solution, which effectively submerged the seedling roots. The six distinct abiotic treatments administered to *S. cereale* plant seedlings included ultraviolet radiation (UV, 70 µW/cm^2^, 220 V, 30 W), flooding (affecting the whole plant), salt (5% NaCl), drought (10% PEG6000), high temperature (40℃), and cold (4℃). For each stress treatment, we established five parallel sets. To analyze the expression patterns, we examined the leaves, roots, and stems of rye seedlings at 0, 1, 4, and 12 h following stress induction. Lastly, considering the presence of diverse hormone response elements within the promoter region of these genes, we conducted three distinct hormone treatments during the flowering stage. These treatments included gibberellin (GA3, 100 µM), auxin (indole-3-acetic acid, IAA, 100 µM), and abscisic acid (50 µM).

### Total RNA extraction, cDNA reverse transcription, and qRT-PCR analysis

Total RNA was extracted from fresh tissues using a plant RNA extraction kit (TianGen RNA Easy Fast Plant Tissue Kit, DP452). The reverse transcription kit was selected from TianGen FastKing One-Step RT-qPCR Kit (SYBR, FP313-01). Primer 5.0 software was used to design qRT-PCR primers for all genes (Table S8). Actin was used as the internal control. The standard expression for SYBR Premix ExTaqII (Takara Bio) was repeated on the CFX96 real-time system (Bio-Rad) for 3 replicates. The total reaction system of qRT PCR was 20 µL, containing 1µL cDNA (100ng/µL^− 1^), 10µL SYBR Green Realtime PCR Master Mix (Takara Bio), 0.5µL forward and reverse primers, and 8µL RNase-free water, respectively. All quantitative primers for genes were analyzed for their practicality through melting curves. The expression of these *bHLH* genes was analyzed using the 2^− (ΔΔCt)^ method [91].

### Statistical analysis

The least significant difference test (LSD) was conducted using significance levels of 0.05 in JMP6.0 software. Origin 2016 (OriginLab Corporation, Northampton, Massachusetts, USA) was used for the histogram drawing. Based on the Pearson correlation program, we defined the correlation coefficient of *ScbHLH* genes using Sigmaplot 12.0 software. The Pearson correlation matrix of the bHLH genes in rye was constructed using R2.11, and network analysis (CNA) was performed using Cytoscape 2.7.0 software. The correlation coefficient was defined at P < 0.05.

### Electronic supplementary material

Below is the link to the electronic supplementary material.

**Supplementary Material 1: Table S1.** List of the 220 *S. cereale* *bHLH* genes identified in this study

**Supplementary Material 2: Table S2.** Analysis and distribution of conserved motifs of bHLH proteins in seven species

**Supplementary Material 3: Table S3.** Cis-regulatory elements in the promoter region of *ScbHLH* genes

**Supplementary Material 4: Table S4.** The tandem duplication clusters of ScbHLH genes

**Supplementary Material 5: Table S5.** The six pairs of segmental duplicates in *S. cereale* bHLH genes

**Supplementary Material 6: Table S6.** One-to-one orthologous relationships between *S. cereale* with other plants

**Supplementary Material 7: Table S7.** Results of Tajima’s D neutrality test

**Supplementary Material 8: Table S8.** Primer sequences for qRT-PCR

**Supplementary Material 9:** Figure S1–S6 in this study

## Data Availability

The entire *Secale cereale* genome sequence information was obtained from the NCBI (National Center for Biotechnology Information) GenBank website, the access number is JADQCU000000000. Rye materials (Weining) used in the experiment were supplied by Prof. Kuiying Li of Anshun University. The datasets supporting the conclusions of this study are included in the article and its additional files.

## Abbreviations

*bHLH*: Basic helix-loop-helix
*ScbHLH*: *Secale cereale bHLH*
qRT-PCR: Quantitative real-time polymerase chain reaction
*AtbHLH*: *Arabidopsis thaliana bHLH*
HMM: Hidden Markov Model
pI: Isoelectric point
CDS: Gene coding sequence
DPA: Days post-anthesis
