# Supplementary material for: Basic helix-loop-helix (bHLH) gene family in rye (Secale cereale L.): genome-wide identification, phylogeny, evolutionary expansion and expression analyses
Source: BMC Genomics. 2024 Jan 17;25:67. doi: 10.1186/s12864-023-09911-3 (PMC10792839; doi:10.1186/s12864-023-09911-3)
Supplement: Supplementary file 9 — Supplementary Material 9: Figure S1–S6 in this study [file 12864_2023_9911_MOESM9_ESM.docx]

**Supplementary Information**

**
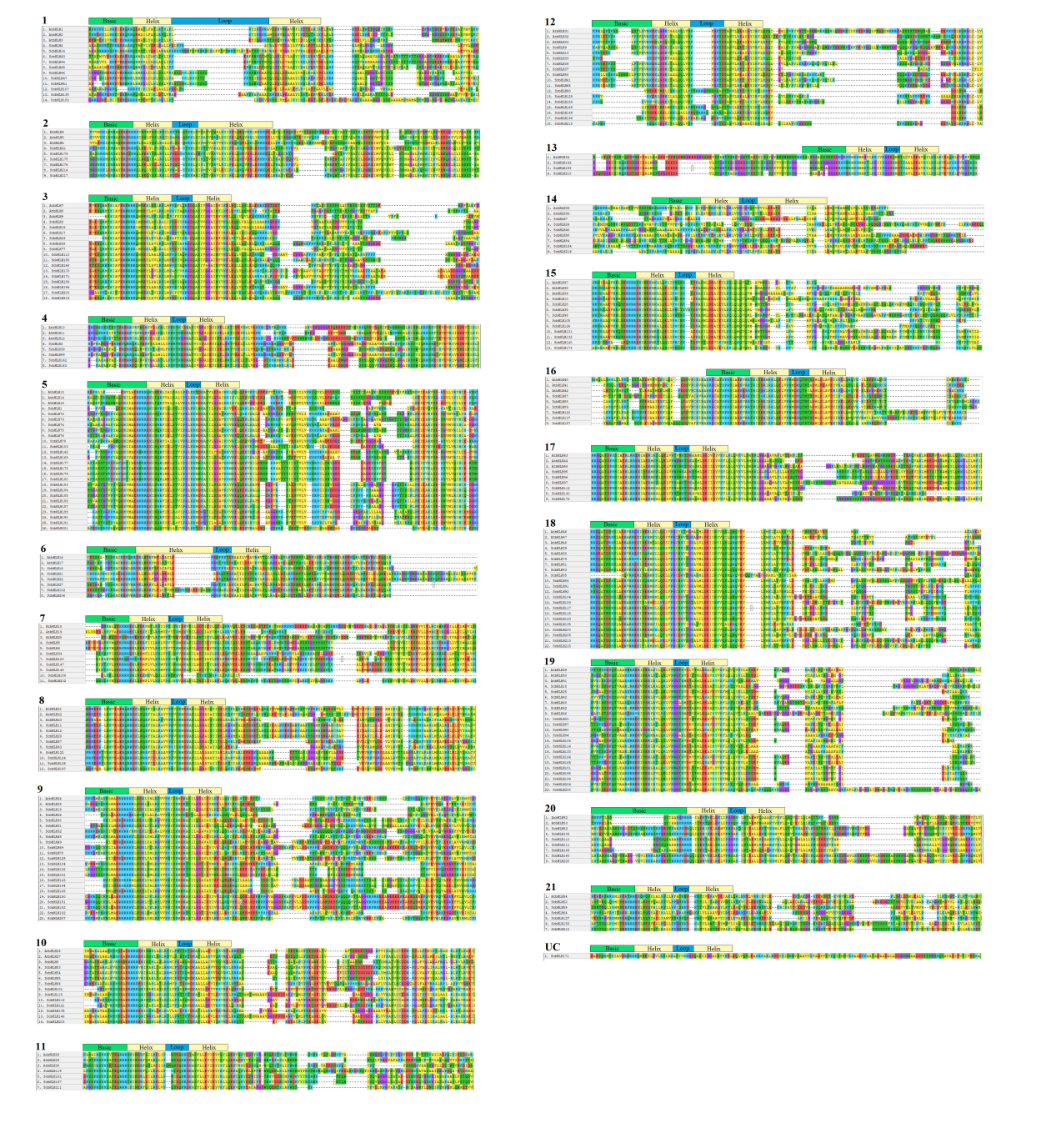
**

**Figure S1:** Multiple sequence alignment of the bHLH domains of the members of ScbHLH and AtbHLH protein family. The scheme at the top depicts the locations and boundaries of the basic, helix, and loop regions in the *bHLH* domain.

**
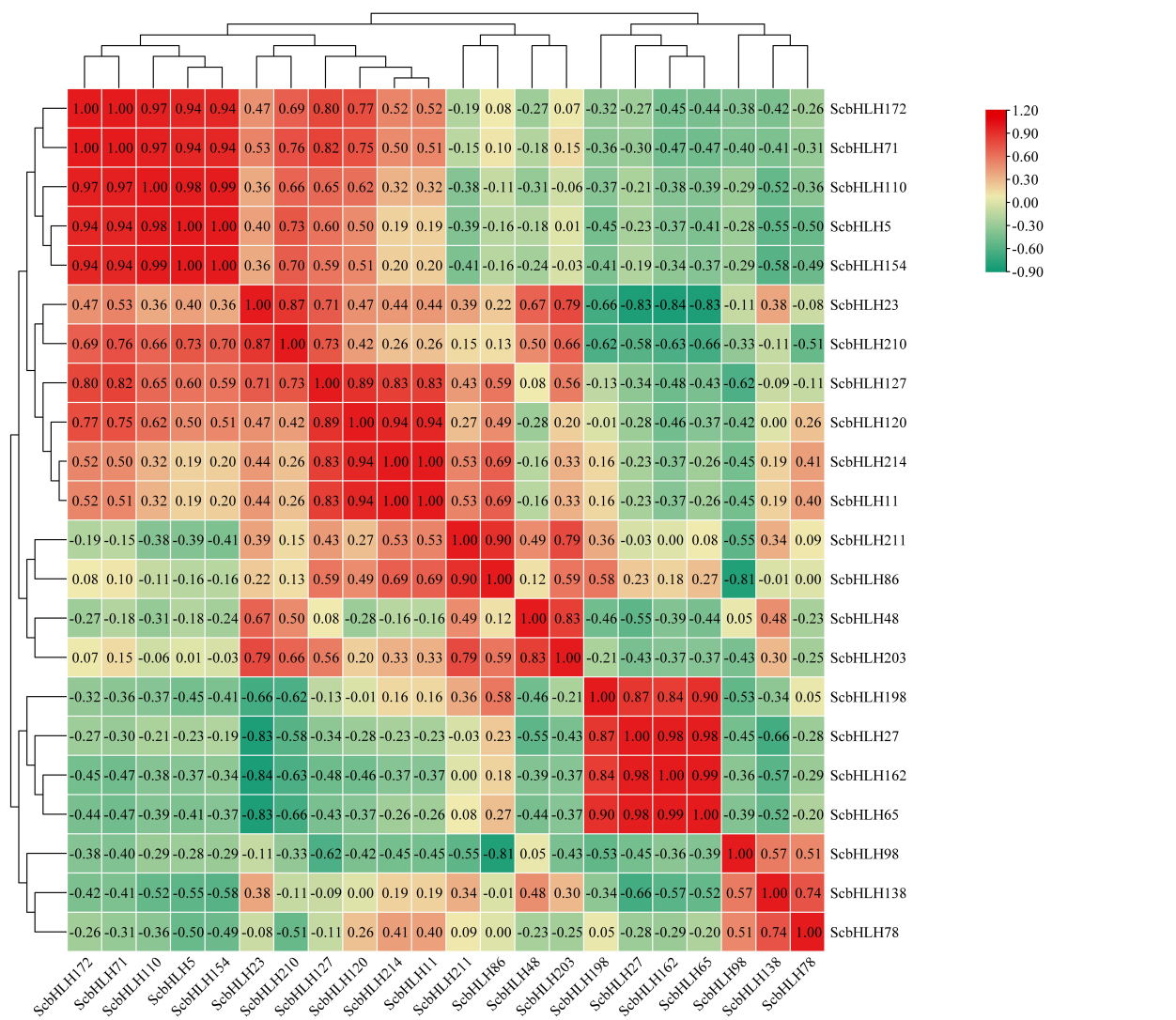
**

**Figure S2:** The correlations of 22 *S. cereale bHLH* genes in several plant organs.


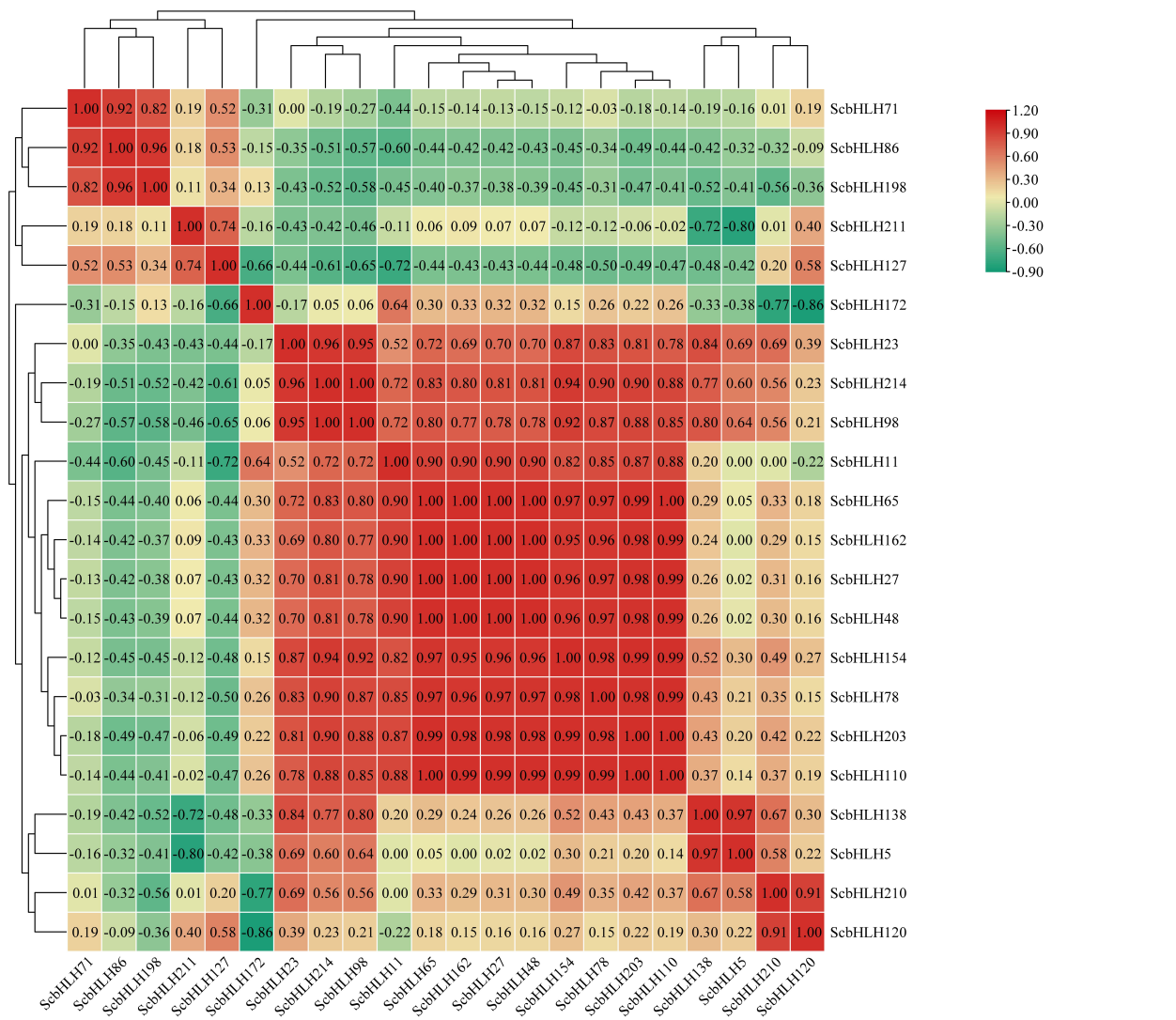


**Figure S3:** The correlations of 22 *S. cereale bHLH* genes during fruit development.


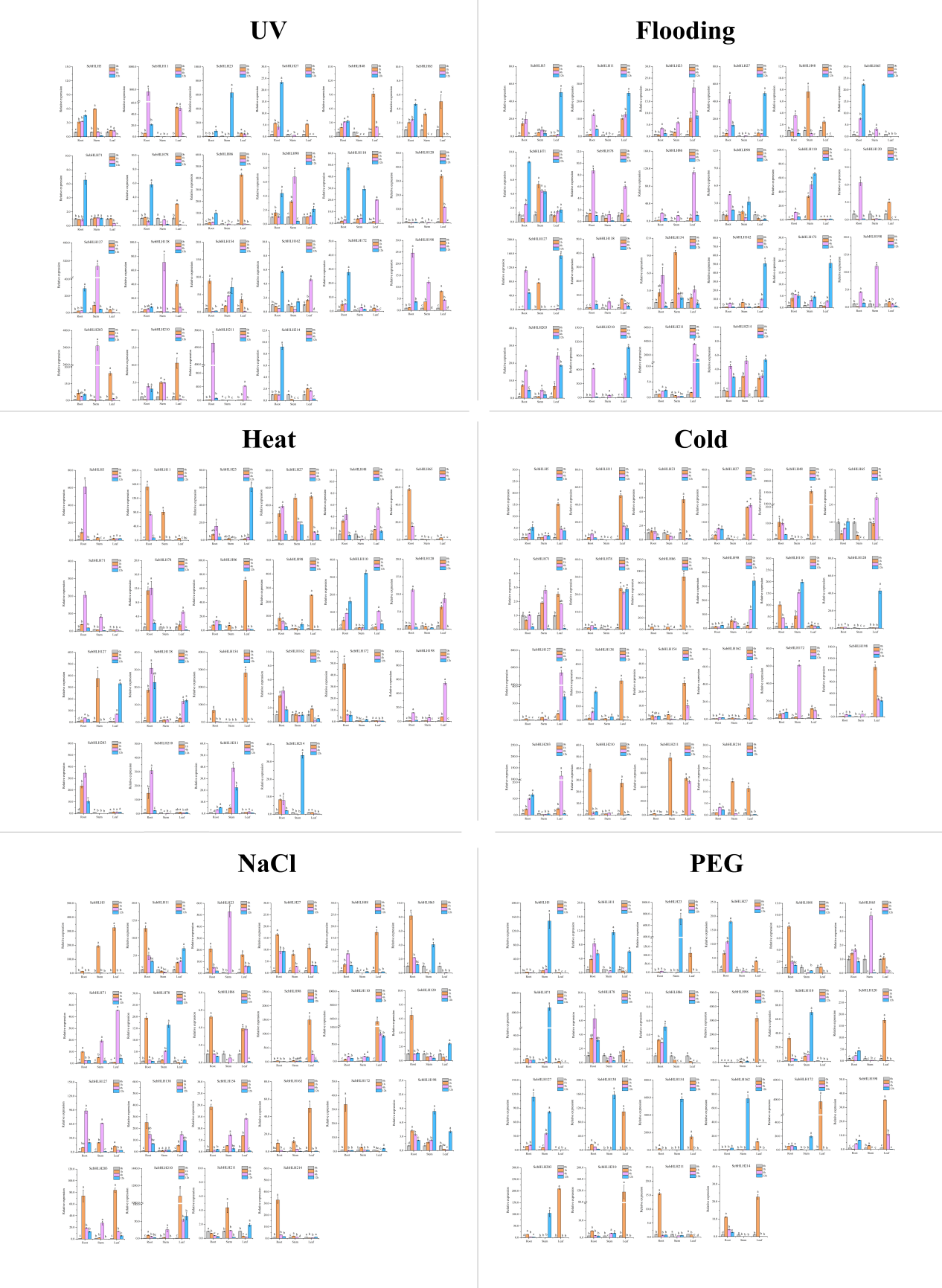


**Figure S4:** Gene expression of 22 *S. cereale bHLH* genes during six abiotic stresses (UV, flooding, PEG, NaCl, heat, and cold) at the seedling stage. The expression patterns of 22 *S. cereale bHLH* genes in leaf, root, and stem organs were examined via qRT-PCR. Error bars were obtained from three measurements. Lowercase letters above the bars indicate significant differences (α = 0.05, LSD) among the treatments.


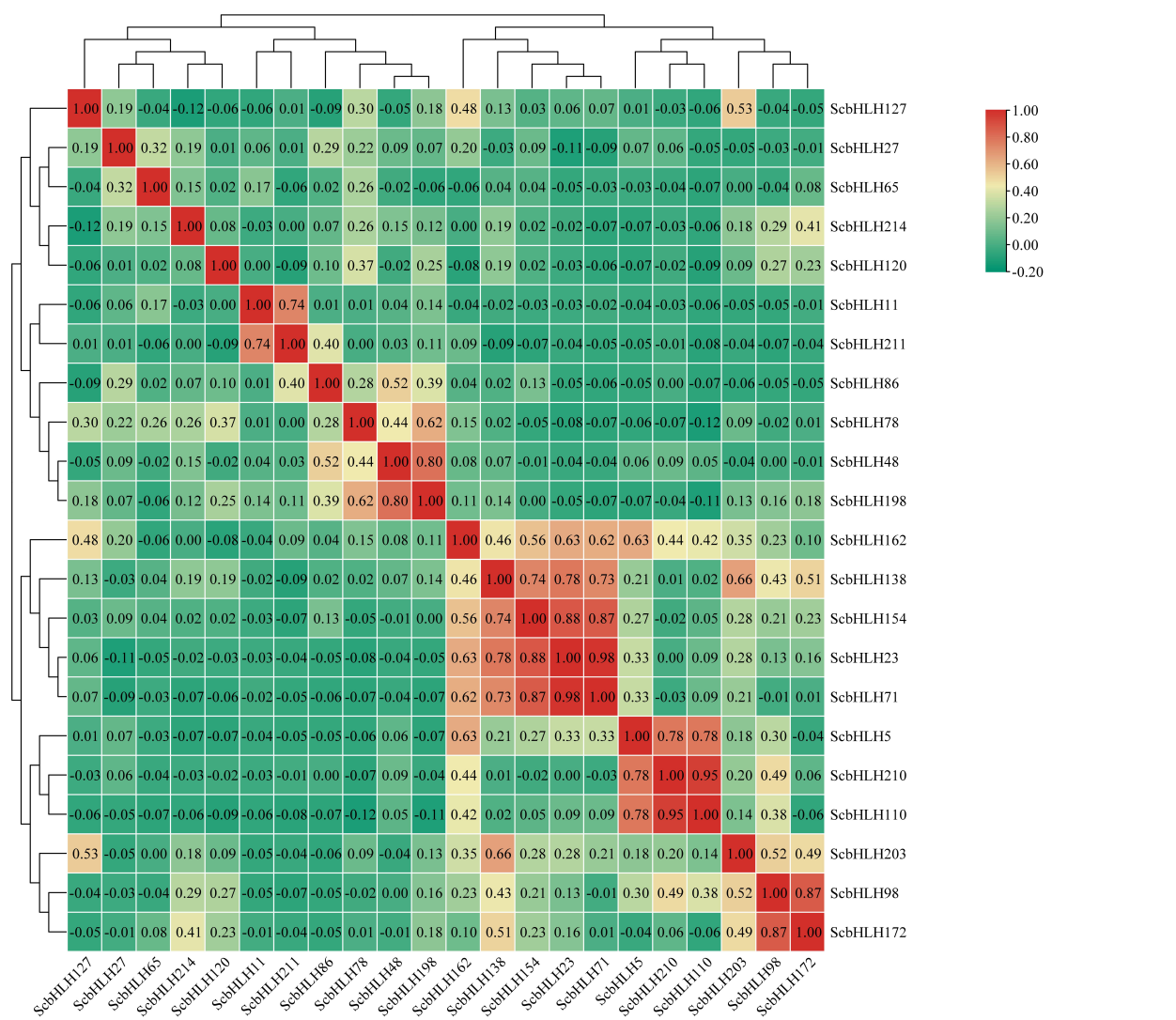


**Figure S5:** The correlations of 22 *S. cereale bHLH* genes in several abiotic stresses.


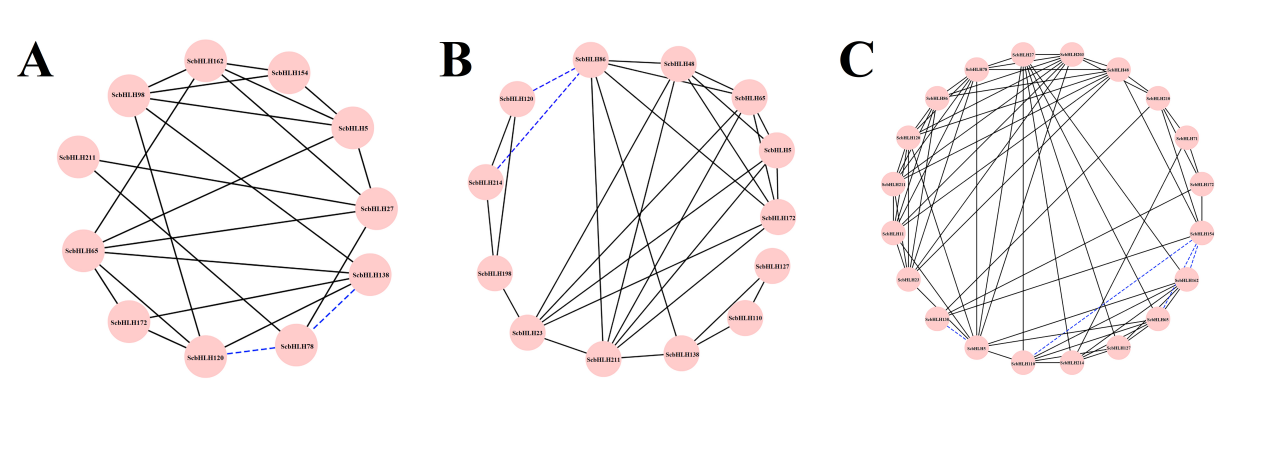


**Figure S6:** Correlation network of the expression of *ScbHLH* family members in fruits treated with different hormones. Among them, A, B, and C are gibberellin, auxin, and abscisic acid respectively.

**Table S1:** List of the 220 rye *bHLH* genes identified in this study.

**Table S2:** Analysis and distribution of conserved motifs of bHLH proteins in seven species.

**Table S3:** Cis-regulatory elements in the promoter region of *ScbHLH* genes.

**Table S4:** The tandem duplication clusters of *ScbHLH* genes.

**Table S5:** The six pairs of segmental duplicates in *S. cereale* *bHLH* genes.

**Table S6:** One-to-one orthologous relationships between *S. cereale* with other plants.

**Table S7:** Results of Tajima's D neutrality test.

**Table S8:** Primer sequences for qRT-PCR.
